# Prevalence of Risk Factors of Retinal Diseases among Patients in Madang Province, Papua New Guinea

**DOI:** 10.1155/2022/6120908

**Published:** 2022-09-05

**Authors:** Bismark Owusu-Afriyie, Isabelle Baimur, Theresa Gende, Thomas Baia

**Affiliations:** ^1^Eye Care Programme, Faculty of Medicine and Health Sciences, Divine Word University, Madang, Papua New Guinea; ^2^Fred Hollows Foundation PNG Inc, Madang, Papua New Guinea

## Abstract

**Purpose:**

To explore the prevalence of risk factors of retinal diseases among patients seeking services from Madang Provincial Hospital Eye Clinic in Papua New Guinea.

**Materials and Methods:**

A hospital-based retrospective study was conducted at the only eye clinic serving the entire Madang province of Papua New Guinea. Purposive sampling was used to obtain data from patients' record cards at the eye clinic from January to June 2021. The data collected included gender, age, presenting visual acuity, blood pressure, blood sugar level, body mass index, smoking habits, and history of cataract surgery. The data was analyzed using the International Business Machines Corporation's Statistical Package for Social Sciences version 21. A *p*-value of <0.05 was considered statistically significant.

**Results:**

Two hundred and fifty-five (255) patients went through diabetes and hypertension screening during the period of the study (January to June 2021). The mean age of the patients was 53.14 ± 11.20 years and there were more males (56.86%) than females. Nearly half of the patients (43.6%) were either visually impaired or blind. More than half (52.6%) had diabetes mellitus. Majority of the patients (73.3%) were hypertensive and more than half (57.0%) of the patients had unhealthy body mass index (BMI <18.525 kg/m^2^ or > 25 kg/m^2^). Overweight was significantly associated with hypertension (*p* < 0.001) and diabetes mellitus (*p* < 0.001). A few of them were smokers or had a history of cataract surgery (13.7% and 2.0%, respectively).

**Conclusion:**

There is a high prevalence of diabetes, hypertension, and overweight among ophthalmic patients in Madang. It is important that measures are put in place to eliminate barriers to health care and to strengthen eye care services in Papua New Guinea.

## 1. Introduction

The retina is the light-sensitive part of the eye and plays significant role in visual function [1–3]. Retinal diseases are the leading cause of irreversible blindness in developed countries and their prevalence is gradually increasing in the developing world [4]. Diseases of the retina such as diabetic retinopathy (DR), hypertensive retinopathy, retinal tear and detachment, epiretinal membrane, macular hole, age-related macular degeneration (AMD), vascular occlusions, and retinitis pigmentosa are significant causes of visual impairment and blindness [5, 6]. For instance, diabetic retinopathy is known to be the main cause of blindness among the working population in the developed world [7, 8] and AMD is the leading cause of blindness among the older population [9–11]. Several studies have reported the prevalence of retinal disorders ranging from 2.42% to 21.02% in different contexts [4, 6, 12, 13].

Therefore, there is a need to identify the risk factors of these diseases among different populations and develop measures to reduce their incidence and progression. Systemic diseases such as hypertension [5, 14] and diabetes mellitus [13, 14], overweight [15, 16], smoking [17, 18], high myopia [19], increased life expectancy (aging population) [1, 5], post-cataract surgery [5, 17], and family history [17, 20] are reported contributing factors for medical retina disorders.

In 2017, Burnett et al. [21] indicated that the prevalence of DR and/or maculopathy in people aged 50 years and above in the National Capital District of Papua New Guinea (PNG) was 46.4%. The rapid assessment of avoidable blindness study also identified posterior segment diseases, DR, and AMD as principal causes of visual impairment and blindness in PNG [22]. More recently, Owusu‒Afriyie et al. [23] indicated that the majority of ophthalmic patients in Madang province (93.6%) sort for eye care services only when the problem affects their eyesight. Yet to date, there is no study to estimate the prevalence of risk factors of retinal morbidities in PNG. Eye care services in PNG are faced with an array of challenges including inadequate and unequal distribution of ophthalmic personnel and services, therefore such information will be useful for training, education, and planning healthcare services to keep life and vision-threatening conditions under control in the country. This study aims to explore the prevalence of risk factors of retinal conditions among ophthalmic patients in Madang province of PNG.

## 2. Materials and Methods

### 2.1. Study Setting

This study was carried out at the Madang Provincial Hospital Eye Clinic in Papua New Guinea. The facility is funded by The Fred Hollows Foundation New Zealand to provide free services and medications to all patients and is one of the main eye care centers in the country. All patients aged 30 years and above undergo diabetes and hypertension screenings as part of the standard procedures at the eye clinic at the time of this study. The facility also provides diabetic retinopathy screening services.

### 2.2. Study Design

This was a hospital-based retrospective study and involved a review of patients' records at the eye clinic from January to June 2021 to obtain data on patients' demographics and risk factors of retinal diseases.

### 2.3. Sampling Technique

Purposive sampling was used in this study. The study involved all available records of patients on diabetes and hypertension screening register at the eye clinic.

### 2.4. Inclusion and Exclusion Criteria

The study included data from all ophthalmic patients attending the clinic from January to June 2021. However, those below 30 years were excluded from the study since the clinic does not include them in diabetes and hypertension screenings.

### 2.5. Study Definitions

The following definitions were used for the purpose of this study [24–30]:

#### 2.5.1. Visual Impairment

- Normal vision: presenting visual acuity ≥6/12 in the better eye.
- Mild visual impairment: presenting visual acuity <6/12 to ≥6/18 in the better eye.
- Moderate visual impairment: presenting visual acuity <6/18 to ≥6/60 in the better eye.
- Severe visual impairment: presenting visual acuity <6/60 to ≥3/60 in the better eye.
- Blindness: presenting visual acuity <3/60 in the better eye.

#### 2.5.2. Hypertension

- Normal blood pressure: systolic pressure of <120 mmHg or diastolic pressure of <80 mmHg.
- Prehypertension: systolic pressure of between 120 and 139 mmHg, or diastolic pressure between 80 and 89 mmHg.
- Hypertension: systolic blood pressure of ≥140 mmHg, or a diastolic pressure of ≥90 mmHg.
- Hypertensive crisis: systolic pressure of >180 mmHg, or a diastolic pressure of >120 mmHg.

#### 2.5.3. Diabetes

Fasting blood sugar level was defined as the blood sugar reading taken at least eight hours after a meal. Random blood sugar level was defined as the blood sugar reading taken at any time of the day regardless of when a meal was had.

- Hypoglycemia: fasting blood sugar level of <4.0 mmol/L or a random blood sugar of below 3.9 mmol/L.
- Normal blood sugar level: a fasting blood sugar level from 4.0 to 5.4 mmol/L or random blood sugar reading of 4.0 to <7.7 mmol/L.
- Prediabetes: fasting blood sugar level of 5.5–6.9 mmol/L or a random blood sugar of 7.7–11.0 mmol/L.
- Diabetes (hyperglycemia): fasting blood sugar ≥7.0 mmol/L or a random blood sugar level ≥11.1 mmol/L.

#### 2.5.4. Body Mass Index

Body mass index (BMI) was defined as a measure of body fat indicated by the person's weight in kilograms per square meter of the person's height (kg/m^2^). It was further classified as follows:

- Very underweight: BMI of <17 kg/m2.
- Underweight: BMI >17 to 18.5 kg/m2.
- Healthy weight: BMI >18.5 to 25 kg/m2.
- Overweight: BMI >25 to 30 kg/m2.
- Obesity: BMI >30 to 35 kg/m2.
- Severe obesity: BMI >35 kg/m^2^.

### 2.6. Ethical Consideration

The study was approved by the Research Ethics Committee of Divine Word University with approval number FRC/MHS/57–21 and permission was granted by the eye clinic's management before data was collected. The study also adhered to the principles of the Declaration of Helsinki.

### 2.7. Data Collection Procedure

Data were collated on a Microsoft Excel spreadsheet and involved a review of diabetes and hypertension screening register and the patients' record cards. Only data on the first visit was extracted and these were the age, gender, weight, height, BMI, blood pressure, and blood sugar level from the screening register. Patient's medical and ocular history, smoking behaviors, visual acuity, and diagnosis were extracted from the patient's record cards. The clinic uses a digital machine (brand: CITIZEN systems) to check the blood pressure and the blood glucose level was taken using ACCU-CHEK glucometer. The BMI was calculated and classified based on the Nutrition Australia BMI chart and guideline that is used at the eye clinic.

### 2.8. Data Management and Analysis

Data were manually entered, cleaned, and edited for inconsistencies. The data was then analyzed using the International Business Machines Corporation's Statistical Package for Social Sciences version 21 (IBM Corp., Armonk, N. Y., USA). Descriptive statistics were computed for all variables. Categorical data were presented as frequencies and percentages, and continuous data as mean ± standard deviation. The chi-square test for independence was used to determine associations between variables. A *p* value of <0.05 was considered statistically significant.

## 3. Results

A total of 477 patients visited the eye clinic during the period under study out of which 255 patients (53.46%) were screened for diabetes and hypertension and thus, met the inclusion criteria for this study.

### 3.1. Demographic Characteristics of Patients

The mean age of the patients was 53.14 ± 11.20 years and ranged from 30 to 90 years. Males who comprised the majority of the patients (56.86%) were significantly older than females (*p*=0.007).  Table 1 shows the age and gender distribution of the patients.

The occupation of most patients (61.2%) was not recorded but of those who did have occupation recorded, the greatest number were farmers (16.1%) and housewives (13.3%). Almost all patients (92.9%) were from the Madang province. A total of 208 (81.6%) patients were dwellers of Madang district which is the administrative capital of Madang province. Morobe province accounted for six patients (2.4%) and the remaining twelve patients (4.7%) were almost equally distributed among eight other provinces in PNG.

### 3.2. Medical and Ocular Health History

More than three-quarters (84.7%) of the patients reported no prevailing or past medical conditions. The most commonly reported conditions were diabetes (3.1%), kidney disease (2.0%), asthma (2.0%), tuberculosis (1.6%), trauma (1.6%), and anemia (1.2%). Similarly, 209 patients (82.0%) had no previous ocular problem but refractive error was commonly reported by 22 (8.6%). Eye injuries and previous cataract surgery were also reported (3.9% and 2.0%, respectively).

### 3.3. Clinical Data and Prevalence of Risk Factors of Retinal Diseases

#### 3.3.1. Smoking Habits

Less than one-quarter (13.7%) of the patients were smokers. There was no significant association between smoking habits and gender (*p*=0.441) or between smoking habits and age group (*p*=0.258).

#### 3.3.2. Category of Visual Impairment

Table 2 shows the distribution of visual impairments and demographics. Almost one-third of the patients (29.0%) had mild visual impairment while moderate and severe visual impairments accounted for less than one-tenth each.

### 3.4. Blood Pressure and Blood Sugar Levels

The mean (±SD) systolic blood pressure was 134.43 ± 22.34 mmHg and that of the diastolic blood pressure was 78.24 ± 12.88 mmHg. More than one-third (35.7%) of patients were prehypertensive and a third (33.3%) had hypertension. There was no significant association between blood pressure and the patient's demographics. Blood sugar levels were determined either as fasting blood sugar (FBS) or random blood sugar (RBS) depending on the time it was measured. The mean FBS was 6.66 ± 3.11 mmol/L, while RBS averaged 8.47 ± 3.91 mmol/L. Seventy-nine patients (31.0%) were prediabetics and a little over one-fifth had diabetes. There was no statistically significant association between blood sugar levels and patients' demographics.  Table 3 illustrates the distribution of blood pressure and blood sugar level among the patients.

### 3.5. Body Mass Index (BMI) of the Patients

The mean BMI was 24.53 ± 5.54 kg/m^2^ (a range between 14 and 46 kg/m^2^). More than half of the patients had an unhealthy BMI. Overweight (22.4%) and underweight (21.2%) were the highest recorded categories of abnormal BMI. There was a statistically significant association between BMI and gender, age, blood sugar level, and blood pressure (see Table 4). Female patients had higher rates of overweight and obesity while males were more likely to be underweight and very underweight patients (*p*=0.001). The highest rate of overweight and obesity was among patients aged 41 to 50 years (*p*=0.001).

### 3.6. Diagnosis and Presence of Retinal Diseases

Almost one-fifth (18.4%) were diagnosed with refractive error. This was similar to the number of patients who had cataracts (17.6%). Only four patients (1.6%) were diagnosed with retinal diseases. Therefore, no associations were analyzed between retinal disorders and the patient's demographic characteristics or risk factors.

## 4. Discussion

Identifying people at high risk of retinal problems is an essential step for early detection and treatment of ocular and visual conditions before the onset of irreversible blindness. This study shines a light on the prevalence of risk factors of retinal diseases among patients seeking eye care services in Madang province of PNG. A study on retinal conditions was previously conducted in PNG but it focused mainly on diabetic retinopathy [21] and it also did not investigate the prevalence of risk factors in the population.

The subjects in this current study were predominantly adults with an average age of 53.14 ± 11.20 years. Many previous studies have identified aging as a risk factor for AMD, DR, and hypertensive retinopathy [1, 2, 17]. The Bhaktapur retina study [5] reported that 52.37% of people aged 60 years and above had retinal disorders in Nepal. In addition, the Aravind comprehensive eye study [4] indicated that increasing age is significantly associated with vitreoretinal diseases. The National Statistical Office of PNG reported that there has been a decrease in the population of those below 15 years (35.6%) and an increase in the working age group and older population (61.9% and 2.6%) [31]. With this increase in the aging population in PNG, it is expected that the incidence of retinal disorders may rise if measures such as health promotion and increased accessibility to eye care services are not put in place early enough to prevent them.

The majority of the patients (84.7%) reported that they had no history of any medical condition. However, the majority of them (73.3%) were either prehypertensive or hypertensive when they were screened during their visits. The hospital-based prevalence of high blood pressure in this study (37.6%) was comparable to a hospital-based multicenter study in ophthalmic clinics in sub-Sahara Africa (37.3%) [32]. Similarly, more than half (52.6%) of the subjects in this study were detected to be either prediabetics or diabetics based on their measured blood glucose levels. Nonetheless, Nkanga et al. [32] reported a slightly higher prevalence of diabetes among Nigerian eye care patients (29.1%) than the estimated prevalence in this study (21.6%). The prevalence of self-reported diabetes (3.1%) was similar to that of a recent cross-sectional study among ophthalmic patients at Madang Provincial Hospital Eye Clinic (3.9%) [23]. The inconsistency between the health history of the patients and the screening outcomes may be due to a lack of accessibility to primary health care services in PNG or a lack of awareness on the side of the patients. Diabetes is the main cause of DR [14, 33]. Hypertension causes hypertensive retinopathy [34, 35] and further contributes to the onset and progression of DR and retinal vascular occlusions [5, 14, 36, 37]. The data from this study indicate that both hypertension and diabetes are major concerns in Madang province. There is therefore the need to increase health promotion activities in the country as well as strengthen the health care system to provide holistic services. Increased screening of the adult population in the country for these conditions will also be helpful. Moreover, the addition of prescreening services to detect hypertension and diabetes among ophthalmic patients, such as what is done at the Madang Provincial Hospital Eye Clinic, is a promising way to identify these risk factors early enough to provide interventions. To make this effective, it is essential that the government and stakeholders of health care in PNG work towards providing adequate facilities in the provinces to treat detected risk factors and monitor complications arising from such screening programs.

A few of the patients reported a history of tuberculosis (1.6%), smoking (13.7%), and previous cataract surgery (2.0%). Tuberculosis is a known cause of posterior segment diseases such as uveitis and choroiditis [38–41] and a history of cataract surgery and smoking are significant risk factors for AMD [17]. Furthermore, Zhang et al. [34] reported that smoking is a risk factor for hypertensive retinopathy among hypertensive patients in Beijing. There is a possibility that the 17.6% diagnosed with cataracts would undergo cataract surgery and this will increase the percentage of patients with a history of cataract surgery, leading to a higher risk of AMD in the study population.

Nearly one-third (31.1%) of the patients were either overweight, obese, or severely obese. Prediabetes was high in overweight, obese, and severely obese subjects (23.1%, 8.9%, and 1.3%, respectively). Diabetes was also high in patients who were overweight (33.3%). The findings support other studies which have reported that there is a high chance of developing diabetes in overweight and obese people [15, 42, 43] and hence, a greater chance of developing diabetic retinopathy. Rustom et al. [16] also indicated that hypertension is associated with overweight among the military population in Bangladesh. Studies have reported unhealthy lifestyles as a cause of overweight and obesity [16, 42]. Early control of weight gain and lifestyle modifications would be helpful in controlling the risk of developing cardiovascular diseases and retinopathies in people with unhealthy BMI [14, 15].

Almost half of the patients (43.6%) were either visually impaired or blind. This prevalence is higher than the prevalence reported in other hospital-based studies in developing countries [44–46]. However, the prevalence of retinal diseases (1.6%) was lower than the prevalence in other developing countries [4, 6, 12, 13].

## 5. Conclusion

In summary, our study underlines the importance of eliminating barriers to health care and strengthening eye care services in PNG. Policymakers and stakeholders need to institute health promotion programs in the country and educate the public about the risk factors and consequences of retinal conditions as well as cardiovascular diseases. There is a need to educate the public on lifestyle modifications such as a healthy diet and regular exercise which could address the high rates of underweight, overweight, and obesity. Stronger advocacy for greater and improved diabetic retinopathy screening programs is also essential in PNG. The lack of information for certain variables of the patients is a limitation on our findings, and the outcome cannot be fully generalized for the entire country. We propose that a population-based study should be carried out to detect and treat diabetes and hypertension in Madang province to prevent complications such as retinopathies and blindness.

## Figures and Tables

**Table 1:** Gender and age distribution of patients (*N* = 255).

		Gender of patients		Total (%)	*p* value
		Male (%)	Female (%)		
Age group of patients	30–40	15	15	30 (11.76)	0.007
	41–50	30	35	65 (25.49)	
	51–60	36	36	72 (28.24)	
	61–70	49	19	68 (26.67)	
	≥71	15	5	20 (7.84)	
Total		145 (56.86)	110 (43.14)	255 (100.00)	

**Table 2:** Distribution of visual impairments among the patients (*N* = 255).

		Frequency (%) of patients with visual impairment							*p* value
		NV	MiVI	MVI	SVI	Blindness	Cannot be categorized^*∗*^	Total	
Gender	Male	23	45	10	11	2	54	145	0.548
	Female	25	29	8	6	0	42	110	
	Total	48	74	18	17	2	96	255	

Age group	30–40	10	7	2	0	1	10	30	<0.001
	41–50	26	16	0	0	0	23	65	
	51–60	8	29	3	10	0	22	72	
	61–70	3	16	11	6	0	32	68	
	≥71	1	6	2	1	1	9	20	
	Total	48 (18.8)	74 (29.0)	18 (7.1)	17 (6.7)	2 (0.8)	96 (37.6)	255	

NV = normal vision, MiVI = mild visual impairment, MVI = moderate visual impairment, SVI = severe visual impairment. ^*∗*^there was no record of the presenting visual acuity of 96 patients and so their level of vision could not be categorized.

**Table 3:** Distribution of blood pressure and blood sugar levels among the patients (*N* = 255).

		Frequency (%) of patients in different categories of hypertension						*p* value
		Normal	Pre-HTN	HTN	HTN crisis	Cannot be categorized^*∗*^	Total	
Gender	Male	23	57	52	6	7	145	0.137
	Female	31	34	33	5	7	110	
	Total	54 (21.2)	91 (35.7)	85 (33.3)	11 (4.3)	14 (5.5)	255 (100.0)	

Age group	30–40	11	7	9	2	1	30	0.231
	41–50	13	30	15	1	6	65	
	51–60	14	26	24	5	3	72	
	61–70	14	23	26	3	2	68	
	≥71	2	5	11	0	2	20	
	Total	54 (21.2)	91 (35.7)	85 (33.3)	11 (4.3)	14 (5.5)	255 (100.0)	

	Frequency (%) of patients in different categories of diabetes							*p* value
		Normal	Prediabetes	Diabetes		Cannot be categorized^*∗*^	Total	

Gender	Male	57	43	30		15	145	0.779
	Female	36	36	25		13	110	
	Total	93 (36.5)	79 (31.0)	55 (21.6)		28 (11.0)	255 (100.0)	

Age group	30–40	11	10	7		2	30	0.484
	41–50	27	20	9		9	65	
	51–60	20	21	20		11	72	
	61–70	26	20	17		5	68	
	≥71	9	8	2		1	20	
	Total	93 (36.5)	79 (31.0)	55 (21.6)		28 (11.0)	255 (100.0)	

HTN = hypertension, ^*∗*^there was no record of the blood pressure of 14 patients and the blood sugar level of 28 patients and so they could not be categorized.

**Table 4:** Body mass index of patients (*N* = 255).

		Frequency (%) of patients in different categories of body mass index								*p* valu*e*
		Very underweight	Under weigh	Healthy	Overweight	Obesity	Severe obesity	Cannot be categorized^*∗*^	Total	
Gender	Male	7	34	65	26	6	3	4	145	0.001
	Female	5	20	28	31	12	1	13	110	
	Total	12 (4.7)	54 (21.2)	93 (3.6)	57 (22.4)	18 (7.1)	4 (1.6)	17 (6.7)	255	

Age group	30–40	2	9	10	4	3	0	2	30	0.001
	41–50	0	9	28	19	7	0	2	65	
	51–60	2	14	20	16	8	3	9	72	
	61–70	7	12	31	15	0	1	2	68	
	≥71	1	10	4	3	0	0	2	20	
	Total	12 (4.7)	54 (21.2)	93 (3.6)	57 (22.4)	18 (7.1)	4 (1.6)	17 (6.7)	255	

Blood sugar level	Normal	4	22	40	18	8	0	1	93	<0.001
	Prediabetes	2	12	35	18	7	1	4	79	
	Diabetes	3	12	12	18	2	3	5	55	
	Cannot be categorized^*∗*^	3	8	6	3	1	0	7	28	
	Total	12 (4.7)	54 (21.2)	93 (3.6)	57 (22.4)	18 (7.1)	4 (1.6)	17 (6.7)	255	

Blood pressure	Normal	6	13	20	10	3	0	2	54	<0.001
	Pre-HTN	4	19	36	20	7	1	4	91	
	HTN	2	18	31	22	8	0	4	85	
	HTN crisis	0	0	3	5	0	2	1	11	
	Cannot be categorized^*∗*^	0	4	3	0	0	1	6	14	
	Total	12 (4.7)	54 (21.2)	93 (3.6)	57 (22.4)	18 (7.1)	4 (1.6)	17 (6.7)	255	

HTN = hypertension ^*∗*^there was no record of the BMI of 17 patients and so they could not be categorized.

## Data Availability

The data used for this study will be made available upon reasonable request from the authors and the Faculty of Medicine and Health Sciences Research Committee (FMHSRC) of Divine Word University, Madang. The chair of the committee can be contacted at ESchuele@dwu.ac.pg.
